# Hip Adduction Asymmetry in Girls with Adolescent Idiopathic Scoliosis

**DOI:** 10.3390/jcm14092864

**Published:** 2025-04-22

**Authors:** Piotr Kurzeja, Tomasz Szurmik, Karol Bibrowicz, Jarosław Prusak, Bartłomiej Gąsienica-Walczak, Katarzyna Ogrodzka-Ciechanowicz

**Affiliations:** 1 Institute of Health Sciences, University of Applied Sciences in Nowy Targ, 34-400 Nowy Targ, Poland; piotrkurzeja@op.pl (P.K.); jaroslaw.prusak@ans-nt.edu.pl (J.P.); bartlomiej.gasienica@ans-nt.edu.pl (B.G.-W.); 2 Faculty of Arts and Educational Science, University of Silesia, 43-400 Cieszyn, Poland; info@orto-med.com.pl; 3 Science and Research Center of Body Posture, Kazimiera Milanowska College of Education and Therapy, 61-473 Poznan, Poland; bibrowicz@wp.pl; 4 Institute of Clinical Rehabilitation, Faculty of Motor Rehabilitation, University of Physical Culture in Krakow, 31-571 Krakow, Poland

**Keywords:** stiffness moiré effect, hip asymmetry, scoliosis

## Abstract

**Background**: The aim of the study was to assess the difference in hip adduction in extended joint position in girls with adolescent idiopathic scoliosis (AIS). **Methods**: The study group consisted of 69 girls aged 9 to 14 years. The observational cross-sectional study involved interview, the clinical examination (body weight measurement, measuring the body height in an upright position, assessment of the alignment of the spinous processes of the thoracic and lumbar vertebrae, assessment of the location of selected anatomical landmarks of the torso), physical examination in which the shape of the ridge surface was analyzed with the use of the photogrammetric method and the moiré effect projection and tests (test of adduction of both hips). **Results**: Significant differences were noted in the values of abduction for the left and right hip. In the studied group, the mean adduction angle for the left hip was 26.3° and only 19.2° for the right hip. The difference was statistically significant (*p* < 0.001). Similarly, significant relationships were noted by the authors with reference to values of the difference in abduction for both hips and the sizes of thoracic (*p* = 00012) and lumbar curvature (*p* < 0.0001). A significant relationship was also noted between the values of lumbar curvature and the size of adduction for the right hip (*p* < 0.0001). **Conclusions**: Abductive contracture of the right hip was noted in the examined girls with AIS. The degree of scoliotic deformity of the lumbar spine is related to the size of the abductive contracture of the right hip joint.

## 1. Introduction

Scoliosis is a three-dimensional deformity of the spine but also of the trunk which may have negative consequences in terms of self-esteem, pain, and possible negative effects related to orthotic or surgical treatment [1]. The condition affects 1–4% of the general population and most of the patients with AIS are healthy and function well until puberty [1]. Initially, AIS may develop asymptomatically, and its consequences may be felt in the subsequent years, significantly affecting the quality of life and causing osteoarticular deformities, pain, and limitations in the function of internal organs [2,3,4]. However, at one point of development of scoliosis, the condition may progress rapidly in a short period of time which may also translate into trunk deformity [5]. As a result, these changes may negatively affect the patient’s self-image [1,6].

There are several known causes of scoliosis, such as congenital scoliosis or neuromuscular scoliosis; however, the most common is adolescent idiopathic scoliosis (AIS), the causes of which are not fully known. There are studies that describe the role of genetics and biomechanics in the development of scoliosis; however, the true cause of this condition remains largely unknown [5,7,8].

One of the theories of the origin of the condition is related to the discoveries and publications of Hans Mau. He observed certain asymmetries in the “body build of newborns and infants” as well as asymmetries in the range of joint movement and described them as the “syndrome of seven contractures” [9,10]. Spinal deformities in idiopathic scoliosis may be closely related to flexion contracture and external rotation of the hip joint.

The abduction contracture of the right hip may be either actual or functional, which is, in fact, a deficiency in adduction of the right hip in relation to the left hip. The consequence of the right hip abduction contracture is a disruption in the child’s biomechanics, manifested in incorrect walking and standing patterns. This may lead to secondary changes: abnormal growth and alignment of the pelvis, sacral bone, lumbar and thoracic spine [9,10].

Any asymmetry during fetal development concerning the position of the baby’s body, asymmetry in joint movement, and the resulting contractures may have a significant effect on hip function. Restricted abduction, mainly of the right hip, may lead to, for example, the problem of hip mobility, and hip joint articulations may be one of the factors that play a role in the etiopathogenesis of scoliosis [11,12,13,14,15]. The position of the fetus with the spine on the left side and the head directed towards the mother’s uterine fundus may have a special effect on the hip range of motion and the spine axis [8]. Asymmetry of body movement during walking and in a static standing position may also play an important part in the development of functional scoliosis. As a result, it may lead to pelvic asymmetry and restricted mobility of the left hip, contributing to scoliosis [16].

Body asymmetry in people with scoliosis may affect lower limbs; however, to date, there have been very few studies documenting the relationship between lower limb asymmetry with scoliosis. Cole et al. [17] noted a significant correlation between the hip ratio (external/internal rotation) and the Cobb angle. When assessing pelvic radiographs of girls with scoliosis, Saji et al. noted an increased but symmetrical femoral angle of inclination [18]. Burwell et al. [19] found asymmetry in the length of left and right femoral bones and bilateral abnormal elongation of the tibia relative to the foot. Karski noted restriction in right hip adduction which correlated with the angle of spine curvature [20].

According to the above theories, the development of idiopathic scoliosis in children can be significantly influenced, for example, by a significant right hip abduction contracture accompanied by a flexion contracture and external rotation contracture, while at the same time, there is a large adduction range of the left hip.

It should also be noted that Karski’s theory, since it was announced in 1995, has caused much controversy among doctors and other specialists involved in the treatment and prevention of postural defects. It significantly varied from the views held at the time and did not receive favorable opinions from the majority of the medical community in Poland. Karski’s theory relates to the method of diagnosis and method of treatment and describes the main causes of idiopathic scoliosis.

Taking this into account and considering also the scarcity of studies on lower limb asymmetry in patients with scoliosis and the controversy referred to above related to the theory, an attempt was made to determine these changes. The aim of the study was to assess the difference in hip adduction in extended joint position in girls with AIS.

## 2. Materials and Methods

### 2.1. Study Design

The study is observational and was conducted in accordance with the Strengthening the Reporting of Observational Studies in Epidemiology (STROBE) Statement: guidelines for reporting observational studies [21]. All data and analysis of results were collected in accordance with the 1964 Helsinki Declaration and its later amendments. Additionally, the study received approval from the Bioethics Committee [68/KBL/OIL/2021], and all consents from parents or legal guardians were obtained.

### 2.2. Setting

The study was carried out in primary schools in the Sląskie, Małopolskie, and Podkarpackie provinces. The study was carried out between March and December 2019.

### 2.3. Participants

The first author qualified for the study 74 girls aged from 9 to 14.

Inclusion criteria:

- age 9–14 years,
- female,
- adolescent idiopathic scoliosis diagnosed by an orthopedist (based on physical examination and X-ray examination with Cobb angle determination)—primary right thoracic curvature,
- no current health dysfunctions that would affect the obtained results (neurological, orthopedic problems, sensory impairments, postural defects),
- written consent of the parent (guardian) to participate in the study,
- written acceptance of the school principals for the examination,
- no communication problems with the examined child.

Exclusion criteria:

- no written consent of the child’s parents or guardians,
- age of the examined child below 9 years and above 14 years,

The study consisted of four parts:

1. Subject examination: age, sociodemographic data.
2. Physical examination:
  - measurement of body mass,
  - measurement of body height in an upright position,
  - assessment of the position of the thoracic and lumbar spine,
  - assessment of body posture: position of the scapular processes and inferior angles of the scapulae, waist triangles, anterior superior and posterior superior iliac spines, and the greater trochanter.

Body mass and height were measured using a verified medical column scale C315.60/150.OW–3—body height measurement range 100–200 cm (UNIWAG—Professional electronic scales, Krakow, Poland). Anthropometric points on the skin were marked with a medical skin marker from Covidien (Medtronic, Minneapolis, MN, USA).

**Figure 1:**
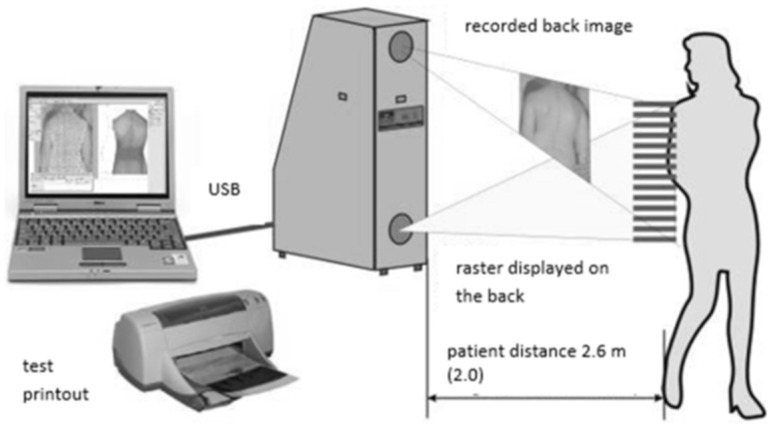
The moiré system [own source].

3. The measurement of the shape of the dorsal surface of the spine was performed using the photogrammetric method and moiré projection (Device for computer assessment of body posture of the MOIRE IV generation system, Wroclaw, Poland) (Figure 1).

4. Functional tests

**Figure 2:**
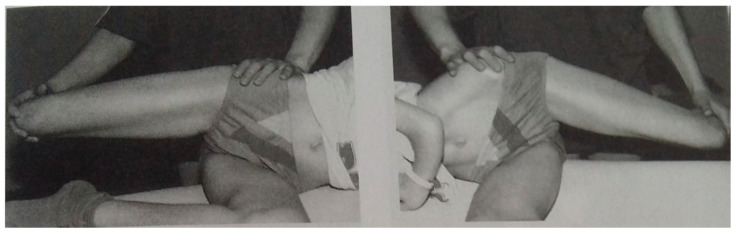
The assessment of the range of hip adduction in extension [22].

To check the limitation of adduction of both hips, the authors used the test proposed by Karski. Test of adduction of both hips (in extension position of joints—modified Ober test) [22]. The test is performed with the patient lying in a lateral position, on the unexamined side, on the edge of the examination table. The patient’s hip joint is in an extended position and with 0° rotation. The examined lower limb is flexed at the knee and is positioned outside the examination table to allow adduction movement. The examiner passively adducts the examined lower limb at the hip joint. Scoliosis becomes apparent first, and it is greatest when adduction of the right hip is 0° and adduction of the left hip is significant, i.e., approx. 40° or even 50°. The people performing the Ober test did not know the previous results of radiological studies. The way of conducting the test is presented in Figure 2.

### 2.4. Outcome Measures

The projection moiré system allows for the assessment of body posture. This technique is based on the refraction of a light beam, which allows for obtaining isolines connecting points of the same height. This technique uses an optical raster placed in the projection device. The image obtained is transmitted by the camera to the computer, which analyzes the data based on a posture assessment program. The result of the analysis is a map of the body surface (a contour image with so-called moiré stripes). The map depends on the illuminated surface (in this case, the back) and the distance of the object from the camera [23,24,25,26,27,28,29,30].

The computerized device for assessing body posture of the MORA 4 system allows for a simple and quick measurement of body posture, which is non-invasive and cost-free.

Using a second camera built into the device, it is possible to capture the entire body of the patient, which reduces the risk of significant posture errors, e.g., head rotation, weight shift to one leg, knee bending, etc.

### 2.5. Scoliosis Measurement

During the examination, the room is darkened. The examined person is wearing underwear and is barefoot. All activities preparing the child for the examination were always performed by the physiotherapist conducting the examination. The physiotherapist marks selected anatomical points on the child’s skin with a washable skin marker. For the examination, the child is positioned in a standing position, and the posterior superior iliac spines (PSIS) are at an equal distance from the camera (the pelvic angle of rotation was 0°). Each child had several to a dozen or so shots taken. From the obtained photos, a single shot was then selected, in which the pelvis was positioned correctly and reflected the patient’s most common posture. The measurement time of one child, including preparation, was about 5 min.

**Figure 3:**
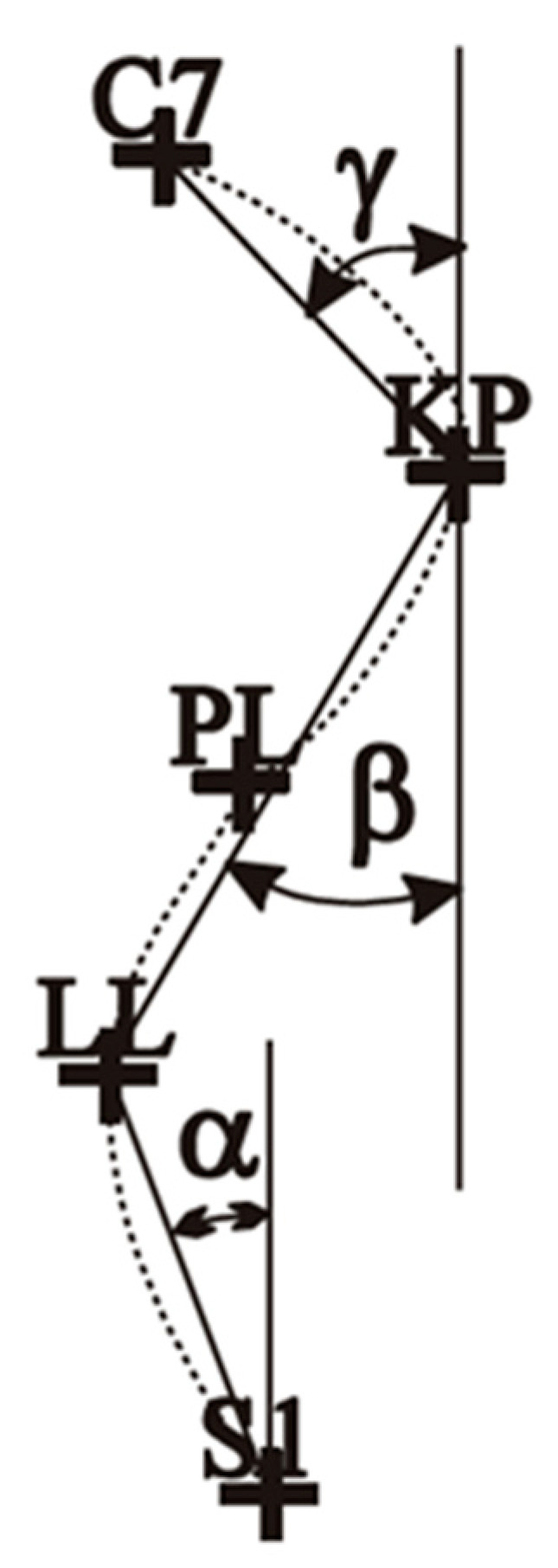
Alfa, Beta, Gamma angles.

In the analysis of the results, three indicators describing changes in the sagittal plane of the spine were used:

- ALFA angle [α]—lumbar-sacral inclination—the angle between the vertical line and the line connecting S1 (the spinous process of the first sacral vertebra) and LL (the peak of lumbar lordosis).
- BETA angle [β]—inclination of the thoracolumbar section. The angle between the vertical line and the line connecting LL (the peak of lumbar lordosis) and KP (the peak of thoracic kyphosis).
- GAMMA angle [γ]—the inclination of the thoracic-upper section. The angle between the vertical line and the inclusion line KP (the peak of thoracic kyphosis) and C7 (the spinous process of the seventh cervical vertebra).
- DELTA angle [δ]—the sum of α, β, and γ (Figure 3).

### 2.6. Statistical Analysis

The statistical analysis of the results of examinations was carried out using MedCalcsoftware (MedCalc^®^ Statistical Software version 23.1.3 (MedCalc Software Ltd., Ostend, Belgium; https://www.medcalc.org; 2025)). In order to assess the distribution of the studied variables, the Shapiro–Wilk test was used. Basic values of descriptive statistics were calculated. The Spearman rank correlation test was used to analyze the correlation between the variables.

## 3. Results

**Figure 4:**
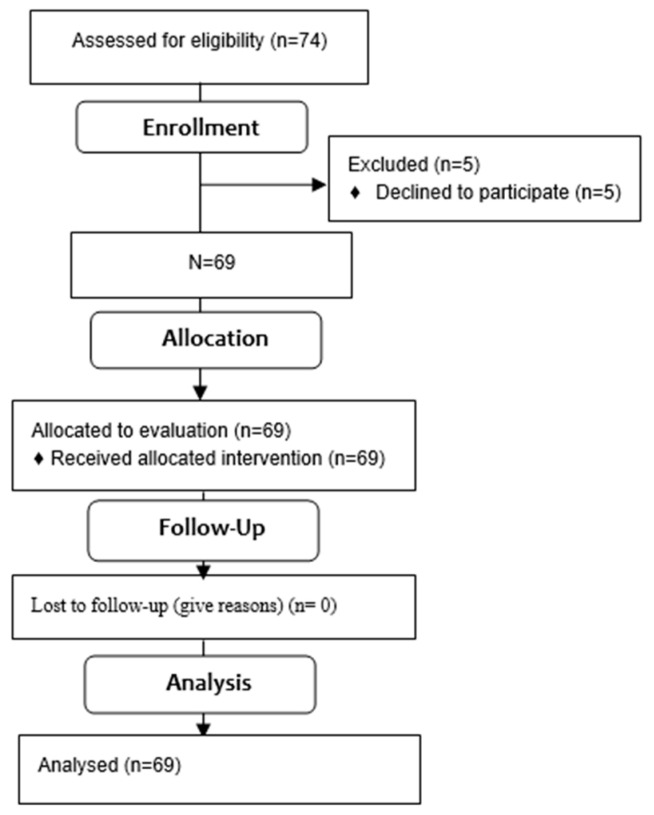
Flow diagram.

The first author qualified for the study 74 girls aged from 9 to 14. Due to a written resignation of the parent/guardian from the child’s participation in the study, 69 girls were qualified for the study in the final stage. Table 1. contains detailed anthropometric data of the examined group. Figure 4. presents the qualification stages.

The values in the adduction test for both hips in the extension position were analyzed. Further on, the relationships between the difference resulting from the size of hip joint adduction and the angle of spine curvature measured using the Cobb method for the lumbar and thoracic spine were determined.

The values of the spine curvatures in the sagittal plane were: angle α 9.10° (SD = 1.14°), angle β 8.54° (SD = 1.01°), angle γ 9.40° (SD = 1.58°), angle δ 27.04° (SD = 3.73°) (Table 2).

The magnitude of the spine curvature measured using the Cobb method was 17.3° (SD = 4.47°) for the thoracic spine and 11.6° (SD = 4.32°) for the lumbar spine (Table 3).

In the comparison of the adduction values for the right and left hip, the difference between the measurements was found to be significant at 9.40° (SD = 1.58°). The analysis of differentiation in the adduction size for the left and right hip in the analyzed group was statistically significant (t = 7.102, *p* < 0.001) (Table 4).

**Figure 5:**
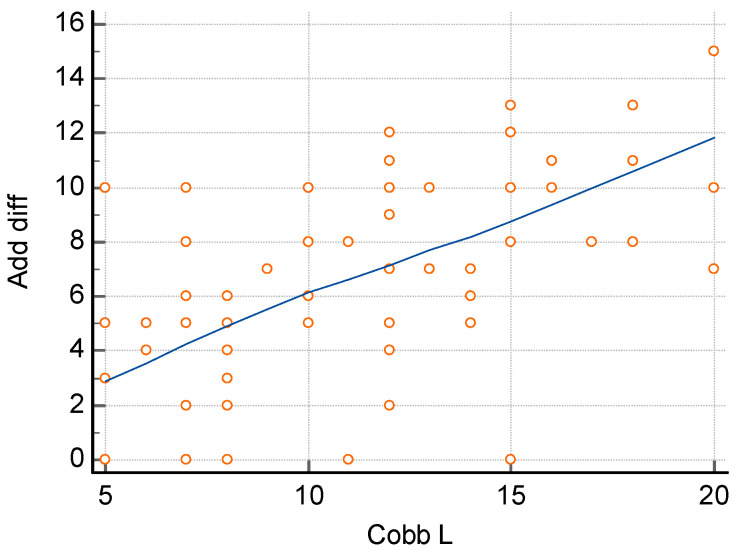
Correlation between the difference in hip adduction and the size of lumbar curvature. R = 0.5792, *p* < 0.0001 *. *—statistically significant.

**Figure 6:**
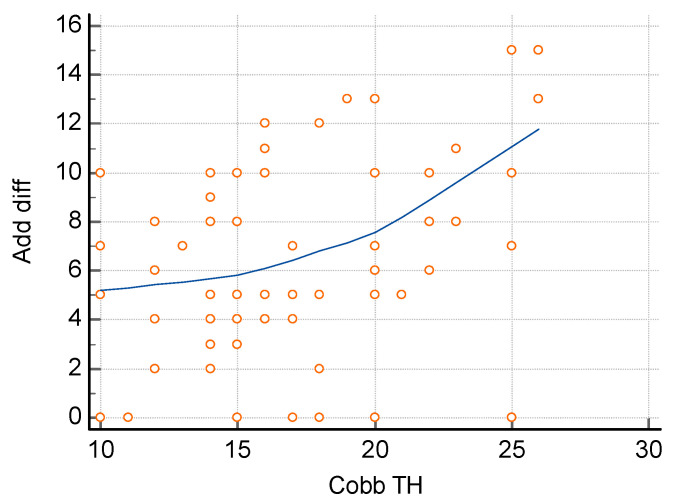
Correlation between the difference in hip adduction and the size of thoracic curvature. R = 0.3816, *p* = 0.0012 *.

**Figure 7:**
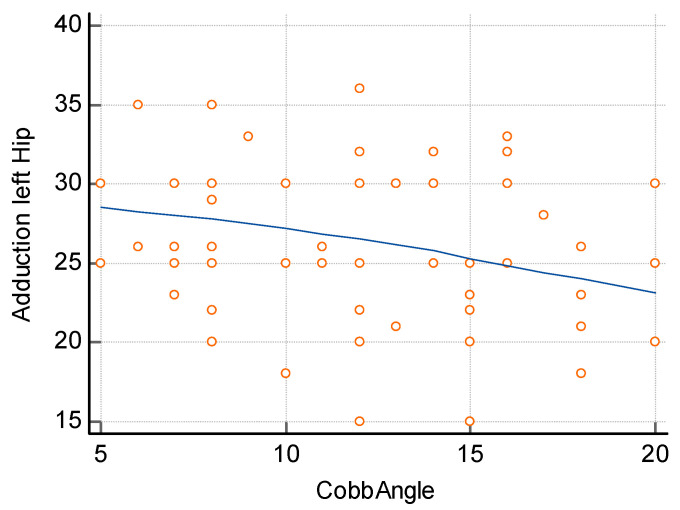
Correlation between left hip adduction and the angle of lumbar curvature. R = −0.3065, *p* = 0.104.

**Figure 8:**
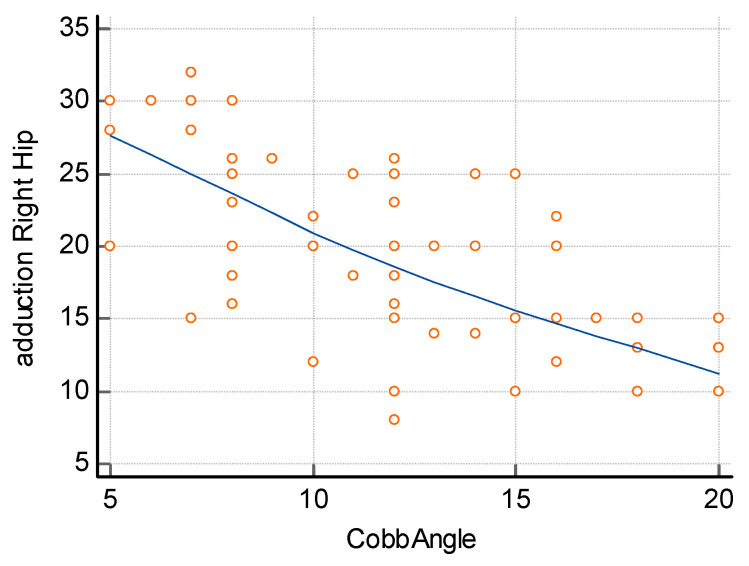
Correlation between right hip adduction and the angle of lumbar curvature. R = −0.6694, *p* < 0.0001 *.

The assessment of the relationship between the difference in the size of hip adduction and the size of curvature, carried out using the Spearman correlation method, showed a significant relationship both for the size of lumbar curvature (Figure 5) and thoracic curvature (Figure 6). In order to determine the relationship between the size of lumbar curvature, an additional analysis of the relationship between the value of curvature and the size of hip adduction was performed separately for the left and right hip. No significant relationship was noted between the size of lumbar curvature and left hip adduction (*p* = 0.104) (Figure 7), whereas a significant relationship was noted between the value of lumbar curvature and the size of adduction for the right hip (*p* < 0.0001) (Figure 8).

## 4. Discussion

The aim of the study was to assess the difference in hip adduction in joint extension in girls with AIS. A total of 69 girls aged 9 to 14 years with diagnosed combined right thoracic and left lumbar scoliosis were qualified for the study. The authors wanted to investigate whether spine deformities in idiopathic scoliosis could be linked to hip contracture. Is there an abductive contracture of the right hip which is actually a deficiency in adduction of the right hip in relation to the left hip? Perhaps the consequence of the right hip abductive contracture could be a disturbance in the child’s biomechanics, manifested as an incorrect walking and standing pattern. This may lead to secondary changes: incorrect growth and alignment of the pelvis, sacral bone, lumbar and thoracic spine. In the authors’ own study and analysis, such relationships were indeed noted. Significant differences were noted in the values of abduction for the left and right hip. In the studied group, the mean adduction angle for the left hip was 26.3° and only 19.2° for the right hip. The difference was statistically significant (*p* < 0.001). Similarly, significant relationships were noted by the authors with reference to values of the difference in abduction for both hips and the sizes of thoracic (*p* = 00012) and lumbar curvature (*p* < 0.0001). A significant relationship was also noted between the values of lumbar curvature and the size of adduction for the right hip (*p* < 0.0001).

Treatment of scoliosis includes surgical and conservative treatment [31,32,33]. However, the prevention of AIS is still an object of study, partly because the etiology and pathogenesis of AIS are still undefined. Although there have been many theories and hypotheses investigating the pathogenesis of AIS, new discoveries still emerge. There are also many methods of treatment and physiotherapy of scoliosis; however, only a small number of them have been recommended by the most important international associations for the prevention and treatment of idiopathic scoliosis, such as the Scoliosis Research Society, the Society on Scoliosis Orthopedic and Rehabilitation Treatment, or the International Research Society of Spinal Deformities. The therapies recommended by the above associations are based on studies and scientific evidence confirming their effectiveness [34,35,36].

One of the many methods of conservative treatment in scoliosis is the method of Tomasz Karski developed in 1995. This method of diagnosing and treatment of scoliosis and its author’s theory about the main causes of idiopathic scoliosis differed significantly from the views held at the time. Karski believed that so-called idiopathic scoliosis develops in growing children as a result of biomechanical disturbances associated with asymmetrical load and discharge of pathological forces in the pelvic area, within the sacropelvic junction and in the lumbosacral and lumbar spine [23]. According to this theory, scoliosis starts “from below”, so the main area which the proposed clinical tests concern is the pelvic area, the hip joints, and the lumbar spine. Tomasz Karski’s theory claims that the cause of scoliosis is abductive contracture, usually in the right hip [37].

In Poland, on the basis of the analysis of the facts and opinions of experts, it was concluded that Karski’s theory and method do not have positive effects on the treatment and rehabilitation of idiopathic scoliosis. A part of the medical community, including the Minister of Health and the national consultant in the field of orthopedics and traumatology of locomotive organs, among others, critically assessed the method of Karski [38].

In light of the above, the authors of this study aimed to verify selected assumptions of Karski’s theory, all the more so that in the available literature, the authors have not found any similar studies apart from the studies of the author of the theory.

According to Karski’s experience, deformities in the locomotive apparatus are caused by asymmetrical shortening of soft tissues, such as tendons, fascia, and joint capsules. Thus, scoliosis develops due to “biomechanical reasons”. This development is associated with asymmetry of movement of the left and right hips [39,40]. Asymmetries in joint movement and asymmetrical “contractures” lead to the development of multiple deformities. Adduction (examining the joint in extension), internal rotation, and extension are the only movements of the right hip joint that are restricted in the etiology of so-called idiopathic scoliosis. These asymmetries are primarily linked to the “syndrome of contractures” [SofC] (as described for the first time by Professor Hans Mau from Tübingen and known in German as the “Siebener Syndrome”) [12,41,42,43].

It should be emphasized again that this point of view is in contradiction with the opinions of many Polish and foreign surgeons [11,44,45,46,47].

Between 1984 and 2022, Karski examined more than 4000 children and adults with scoliosis. Between 1984 and 2009, this study group included children from the Department of Pediatric Orthopedics and Rehabilitation at the Medical University of Lublin, Poland. In addition, Karski examined and treated children and adults in an Outpatient Clinic from 1984 to 2022 [48]. After 11 years of research, Karski found that children with scoliosis showed dissimilarity in hip adduction in extended joint position and, in some cases, also asymmetry of internal hip rotation. Specifically, adduction in the right hip was restricted [48]. Over time, Karski reached a conclusion that the causes of scoliosis are not related “directly to the contracture in right hip abduction” but are linked to “the function resulting from this contracture”, i.e., standing and walking [48]. He also noted that there are additional causes in the development of scoliosis related to certain changes in body anatomy and function related to the central nervous system [49,50,51]. Reduced mobility of right hip adduction or even abduction in joint extension may be the cause of potential “functional influence” on the development of scoliosis. The restricted movement of the right hip may be due to the fact that in 90–97% of cases position of the fetus is on the left side of the mother’s uterus, and in some circumstances in the “prenatal” period, it may lead to the syndrome of contractures and deformities according to Hans Mau [10,49,52,53,54].

More than two thousand years have passed since scoliosis was first described and treated (Hippocrates), and the term “scoliosis” was first used (Claudius Galenus). Until now, however, the etiology of this condition has not been clearly determined. Perhaps changes related to the asymmetry of a child’s anatomy (as early as in newborns and infants), followed by the changes related to the asymmetry of movement in many joints—but what is particularly important—asymmetry in hip movement, are in some way responsible for the intensification of changes leading to spinal deformity (scoliosis).

In their own studies, the authors have confirmed the differences in adduction for the right and left hip in girls with idiopathic scoliosis. This deficiency in adduction of the right hip in relation to the left hip may contribute to the asymmetry of body movement during walking and body asymmetry during standing.

As idiopathic scoliosis is characterized by a complex pathomechanism, it seems advisable to assess each patient individually, taking into account, among other things, potentially existing contractures within the trunk and hip joints. Such an assessment will no doubt help to draw up a comprehensive treatment plan necessary for conservative treatment.

### Study Limitation

This study is not without its limitations. The authors of this article intend to continue their research on a much larger group of girls as well as boys. They realize that their functional assessment of the patients has not been comprehensive. The next stage of the study may be to evaluate the effect of pelvic asymmetry on changes in angles of spine curvature and to assess the contraction of the abductor muscles of the hip joint.

## 5. Conclusions

Abductive contracture of the right hip was noted in the examined girls with AIS. The degree of scoliotic deformity of the lumbar spine is related to the size of the abductive contracture of the right hip joint.

## Figures and Tables

**Table 1:** Data of the observation group.

	Mean	SD	Min–Max
Age [yrs]	11.1	1.12	9.1–14.2
Body weight [kg]	42.2	4.81	34–56
Height [cm]	147.8	7.05	132–162

**Table 2:** Values of spine curvatures in the sagittal plane.

Angle (o)	x ± SD	Min–Max
α	9.10 ± 1.14	6.8–12.3
β	8.54 ± 1.01	5.9–10.6
γ	9.40 ± 1.58	6.2–12.5
δ	27.04 ± 3.73	21.8–33.8

α—alpha angle—the inclination of the lumbosacral segment; β—beta angle—the inclination of the thoracic-lumbar segment; γ—gamma angle—the slope of the upper thoracic segment; δ—the sum of α, β, and γ; x—mean; SD—standard deviation.

**Table 3:** Angular values of curvatures according to Cobb in lumbar and thoracic spine (°).

Cobb (°)	x ± SD	Min–Max
Th	17.3 ± 4.47	10–26
L	11.6 ± 4.32	5–20

Th—angular curvature according to Cobb in the thoracic spine; L—angular curvature according to Cobb in the lumbar spine.

**Table 4:** Values of hip adduction angle (°).

	x ± SD	Min–Max	t	*p*
Add left (°)	26.30 ± 4.91	15–36	7.10	*p* < 0.001
Add right (°)	19.20 ± 6.61	8–32		
Add diff. (°)	9.40 ± 1.58	0–15		

Add left—value of the adduction angle of the left hip; Add right—value of the adduction angle of the right hip; Add diff.—difference in values of adduction angles of both hips.

## Data Availability

The minimal data set is contained within our paper.

## References

[1] Cheng J.C., Castelein R.M., Chu W.C., Danielsson A.J., Dobbs M.B., Grivas T.B., Gurnett C.A., Luk K.D., Moreau A., Newton P.O. (2015). Adolescent idiopathic scoliosis. Nat. Rev. Dis. Primers.

[2] Calvo-Muñoz I., Gómez-Conesa A., Sánchez-Meca J. (2013). Prevalence of low back pain in children and adolescents: A meta-analysis. BMC Pediatr..

[3] Angarita-Fonseca A., Boneth-Collante M., Ariza-Garcia C.L., Parra-Patiño J., Corredor-Vargas J.D., Villamizar-Niño A.P. (2019). Factors associated with non-specific low back pain in children aged 10–12 from Bucaramanga, Colombia: A cross-sectional study. J. Back. Musculoskelet. Rehabil..

[4] Jasiewicz B., Rozek K., Kurzeja P., Daszkiewicz E., Ogrodzka-Ciechanowicz K. (2022). The Influence of Surgical Correction of Idiopathic Scoliosis on the Function of Respiratory Muscles. J. Clin. Med..

[5] Negrini S., Donzelli S., Aulisa A.G., Czaprowski D., Schreiber S., de Mauroy J.C., Diers H., Grivas T.B., Knott P., Kotwicki T. (2018). 2016 SOSORT guidelines: Orthopaedic and rehabilitation treatment of idiopathic scoliosis during growth. Scoliosis Spinal Disord..

[6] Du Q., Zhou X., Li J.A., He X.H., Liang J.P., Zhao L., Yang X.Y., Chen N., Zhang S.X., Chen P.J. (2015). Quantitative ultrasound measurements of bone quality in female adolescents with idiopathic scoliosis compared to normal controls. J. Manip. Physiol. Ther..

[7] Schlösser T.P., van der Heijden G.J., Versteeg A.L., Castelein R.M. (2014). How ‘idiopathic’ is adolescent idiopathic scoliosis? A systematic review on associated abnormalities. PLoS ONE.

[8] Gorman K.F., Julien C., Moreau A. (2012). The genetic epidemiology of idiopathic scoliosis. Eur. Spine J..

[9] Mau H. (1979). Zur Ätiopathogenese von Skoliose. Hüftdysplasie und Schiefhals im Säuglingsalter. Z. Orthop..

[10] Mau H. (1982). Die Atiopatogenese der skoliose, bücherei des orthopäden. Enkeverlag Stuttg..

[11] Burwell R.G., Dangerfield P.H., Burwell R.G., Dangerfield P.H., Lowe T.G., Margulies J.Y. (2000). State of the Art Reviews: Spine. Etiology of Adolescent Idiopathic Scoliosis: Current Trends and Relevance to New Treatment Approaches.

[12] Green N.E., Griffin P.P. (1982). Hip dysplasia associated with abduction contracture of the contralateral hip. JBJS.

[13] Ogilvie J.W., Braun J., Argyle V., Nelson L., Meade M., Ward K. (2006). The search for idiopathic scoliosis genes. Spine.

[14] Karski T. (2002). Etiology of the So-Called ‘Idiopathic Scoliosis’. Biomechanical Explanation of Spine Deformity. Two Groups of Development of Scoliosis. New Rehabilitation Treatment. Possibility of Prophylactics, Studies in Technology and Informatics, Research into Spinal Deformities 4.

[15] Karski T. (2023). Biomechanical etiology of the so-called idiopathic scoliosis: Principles of causal prophylaxis, indications to new therapy. Persp. Recent. Adv. Med. Res..

[16] Karski T. (2022). Biomechanical Etiology of the So-Called Idiopathic Scoliosis (Adolescent Idiopathic Scoliosis [AIS])—Observations in Years 1984–2022 and in Points. CPQ Orthop..

[17] Cole A., Burwell R.G., Jacobs K.J. (1990). Hip rotation, knee rotation and femoral anteversion in healthy subjects and in children with adolescent idiopathic scoliosis: Relation of hip rotation to lateral spinal curves. Clin. Anat..

[18] Saji M.J., Upadhyay S.S., Dorth D.M., Leong J.C. (1995). Y: Increased femoral neck-shaft angles in adolescent idiopathic scoliosis. Spine.

[19] Burwell R.G., Aujla R.K., Freeman B.J.C., Dangerfield P.H., Cole A.A., Kirby A.S., Pratt R.K., Webb J.K., Moulton A. (2006). Patterns of extra-spinal left-right skeletal asymmetries and proximo-distal disproportion in adolescent girls with lower spine scoliosis: Ilio-femoral length asymmetry & bilateral tibial/foot length disproportion. Stud. Health Technol. Inform..

[20] Karski T., Kalakucki J., Karski J. (2006). Syndrome of contractures (according to Mau) with the abduction contracture of the right hip as causative factor for development of the so-called idiopathic scoliosis. Stud. Health Technol. Inform..

[21] von Elm E., Altman D.G., Egger M., Pocock S.J., Gøtzsche P.C., Vandenbroucke J.P. (2008). Initiative STROBE. The strengthening the reporting of observational studies in epidemiology (STROBE) statement: Guidelines for reporting observational studies. J. Clin. Epidemiol..

[22] Karski J., Karski T. (2013). So-Called Idiopathic Scoliosis: Diagnostic Tests: Examples of Children Incorrect Treated: New Therapy by Stretching Exercises and Results. J. Nov. Physiother..

[23] Karski T. (2003). So-Called Idiopathic Scoliosis—Etiology, Risk Recognition, New Rehabilitation Treatment, Prevention.

[24] Drzał-Grabiec J., Truszczyńska A., Tarnowski A., Płaszewski M. (2015). Comparison of parameters characterizing lumbar lordosis in radiograph and photogrammetric examination of adults. J. Manip. Physiol. Ther..

[25] Leroux M.A., Zabjek K., Simard G., Badeaux J., Coillard C., Rivard C.H. (2000). A noninvasive anthropometric technique for measuring kyphosis and lordosis: An application for idiopathic scoliosis. Spine.

[26] Pruijs J.E.H., Keessen W., Van der Meer R., Van Wieringen J.C. (1995). School screening for scoliosis: The value of quantitative measurement. Eur. Spine J..

[27] Ruggerone M., Austin J.H. (1986). Moiré topography in scoliosis. Correlations with vertebral lateral curvature as determined by radiography. Phys. Ther..

[28] Walicka-Cupryś K., Wyszyńska J., Podgórska-Bednarz J., Drzał-Grabiec J. (2018). Concurrent validity of photogrammetric and inclinometric techniques based on assessment of anteroposterior spinal curvatures. Eur. Spine J..

[29] Komeili A., Westover L., Parent E.C., El-Rich M., Adeeb S. (2015). Monitoring for idiopathic scoliosis curve progression using surface topography asymmetry analysis of the torso in adolescents. Spine J..

[30] Bibrowicz K., Szurmik T., Ogrodzka-Ciechanowicz K., Hudakova Z., Gąsienica-Walczak B., Kurzeja P. (2023). Asymmetry of the pelvis in Polish young adults. Front. Psychol..

[31] Zhang H.Q., Gao Q.L., Lei G.E., Wu J.H., Liu J.Y., Guo C.F., Liu S.H., Lu S.J., Li J.S., Yin X.H. (2012). Strong halo-femoral traction with wide posterior spinal release and three dimensional spinal correction for the treatment of severe adolescent idiopathic scoliosis. Chin. Med. J..

[32] Bin Y.U., Zhang J.G., Qiu G.X., Lu W.C., Wang Y.P., Shen J.X., Qi F.E.I., Li Q.Y., Weng X.S. (2010). Selective anterior thoracolumbar/lumbar fusion and instrumentation in adolescent idiopathic scoliosis patients. Chin. Med. J..

[33] Li Q.Y., Zhang J.G., Qiu G.X., Wang Y.P., Shen J.X., Zhao Y., Li S.G., Yu B., Wang X., Weng X.S. (2010). Primary effect of dual growing rod technique for the treatment of severe scoliosis in young children. Chin. Med. J..

[34] Bettany-Saltikov J., Parent E., Romano M., Villagrasa M., Negrini S. (2014). Physiotherapeutic scoliosis-specific exercises for adolescents with idiopathic scoliosis. Eur. J. Phys. Rehabil. Med..

[35] Berdishevsky H., Lebel V.A., Bettany-Saltikov J., Rigo M., Lebel A., Hennes A., Romano M., Białek M., M’hango A., Betts T. (2016). Physiotherapy scoliosis-specific exercises—A comprehensive review of seven major schools. Scoliosis Spinal Disord..

[36] Bettany-Saltikov J., Parent E., Romano M., Villagrasa M., Negrini S. (2021). Adolescent idiopathicscoliosis: Review of conservative treatment with physiotherapy scoliosis specific exercises. Int. J. Health Sci. Res..

[37] Karski T., Karski J., Karska K. (2023). Syndrome of Contractures and Deformities (SofCD): Dysplasia of Hips, Varus Deformity of Shanks, Wry Neck, So-Called Idiopathic Scoliosis Causes Clinic Prophylaxis Therapy. J. Ortho Sci. Res..

[38] Kopacz E. Letter from the Minister of Health Ewa Kopacz, Response to Interpellation No. 18054. https://www.sejm.gov.pl/.

[39] Bensoussan L., Viton J.M., Barotsis N., Delarque A. (2008). Evaluation of patients with gait abnormalities in physical and rehabilitation medicine settings. J. Rehabil. Med..

[40] Jones M.A., Grotewiel M. (2011). Drosophila as a model for age-related impairment in locomotor and other behaviors. Exp. Gerontol..

[41] Heikkilä E. (1984). Congenital dislocation of the hip in Finland. An epidemiologic analysis of 1035 cases. Act. Orthop. Scand..

[42] Hensinger R.N. (1979). Congenital dislocation of the hip. Clin Symp..

[43] Howorth B. (1977). The etiology of the congenital dislocation of the hip. Clin Orthop..

[44] Lowe T., Lawellin D., Smith D., Price C., Haher T., Merola A., O’Brien M. (2002). Platelet calmodulin levels in adolescent idiopathic scoliosis. Spine.

[45] Malawski S. (1994). Own Principles of the Therapy of Beginnings Scoliosis in Actually Point of View to Etiology and Pathogenesis of Scoliosis. Chir. Narz. Ruchu. Ortop. Pol..

[46] Sevastik J., Diab K. (1997). Studies in Technology and Informatics, Research into Spinal Deformities 1.

[47] Zarzycki D., Skwarcz A., Tylman D., Pucher A. (1992). Natural history of lateral curvature of the spine. Chir. Narz. Ruchu. Ortop. Polska..

[48] Karski T. (2022). History of Discoveries of Biomechanical Etiology of the So-Called Idiopathic Scoliosis (Adolescent Idiopathic Scoliosis [AIS]) In Dates And “Think Over”/Meditations. Rules of Therapy. Int. J. Ortho Res..

[49] Vlach O., Rouchal T., Neubauer M. (2003). The Etiology of the So-Called Idiopathic Scoliosis. The New Rehabilitation Treatment. Prophylaxis.

[50] Tomaschewski R., Popp B. (1992). Die Funktionelle Behandlung der Beginnendeni Diopathischen Skoliose.

[51] Tibor V., Karski T. (2000). The Etiology of the So-Called Idiopathic Scoliosis. Progress and Fixation of the Spine Disorders. The Prophylaxis and Principles of the New Rehabilitation Treatment.

[52] Karski T. (1997). Hip abductor contracture as a biomechanical factor in the development of the so-called „idiopathic scoliosis”. Explanation of the etiology. Ann. Univ. Mariae Curie Sklodowska Med..

[53] Karski T. (2019). Biomechanical etiology of the so-called Idiopathic Scoliosis-New classification; Rules of therapy and causal prophylaxis. Int. J. Spine Res..

[54] Karski T. (2021). Minimal Brain Dysfunction. Children and adults. Clinical and Psychological Symptoms. Examples of pathology. Rules of Therapy. Int. J. Ortho Res..

